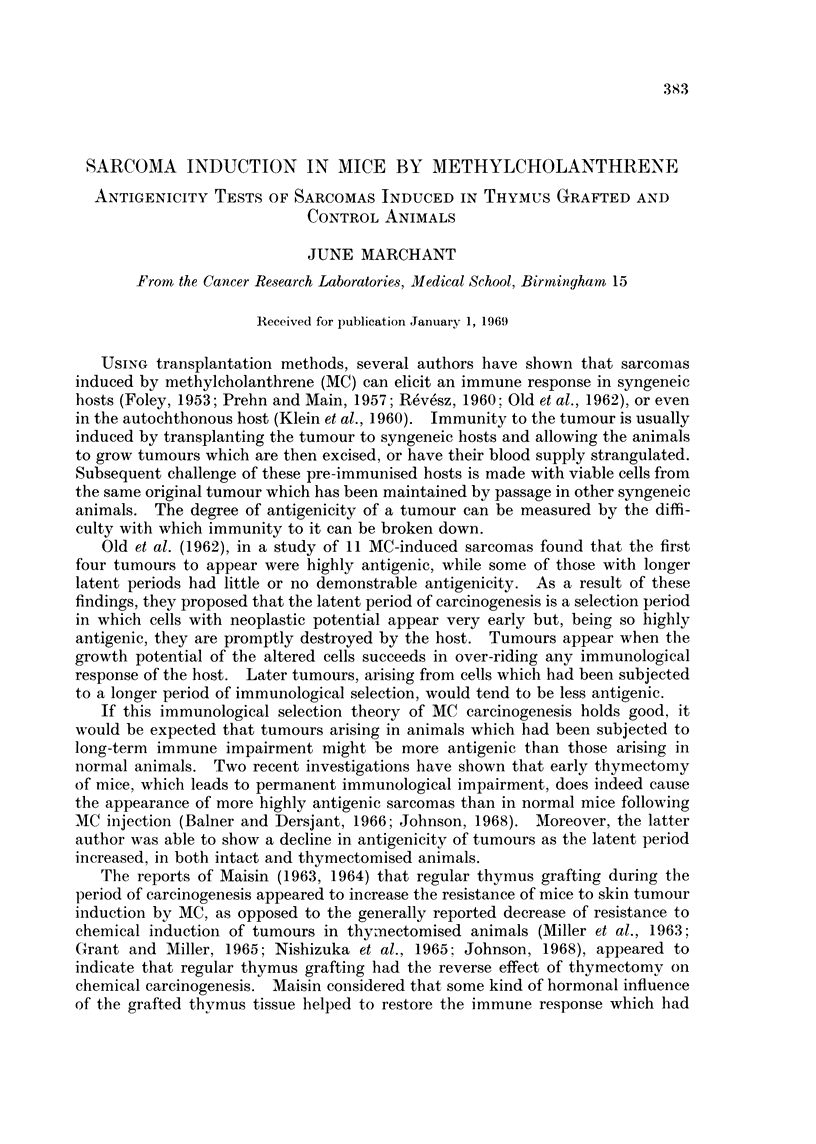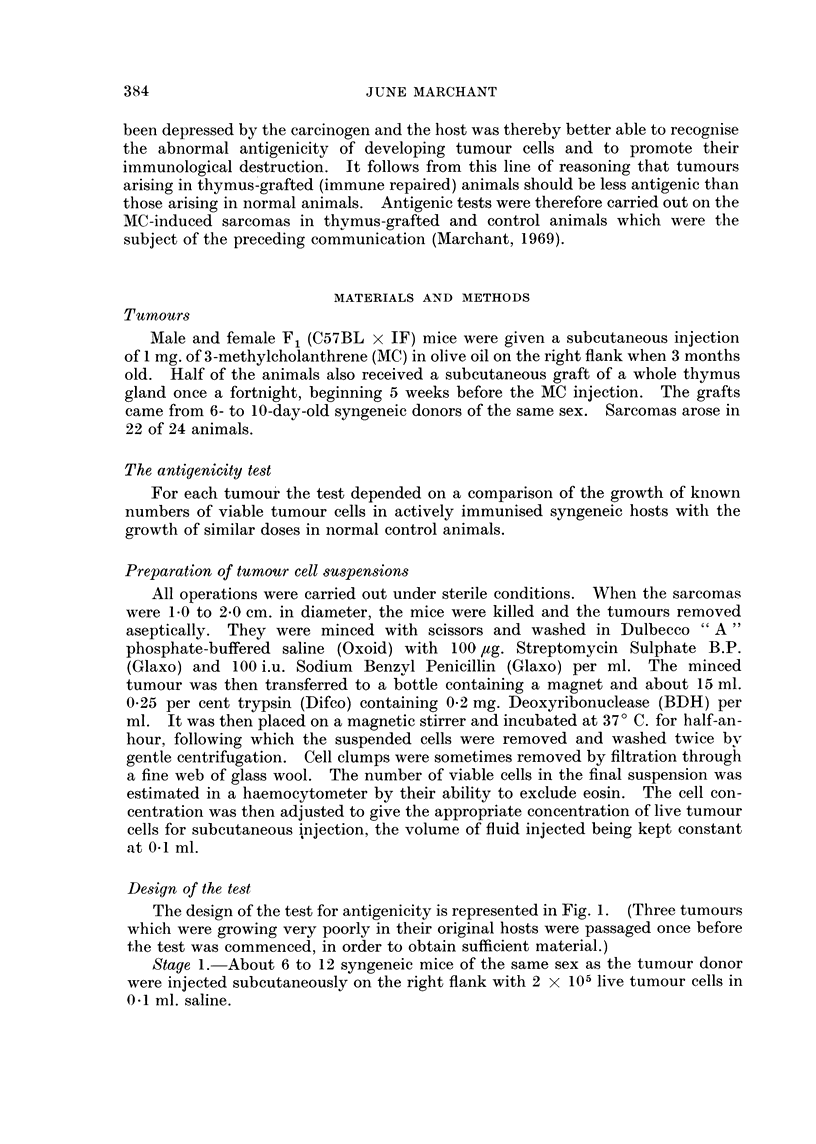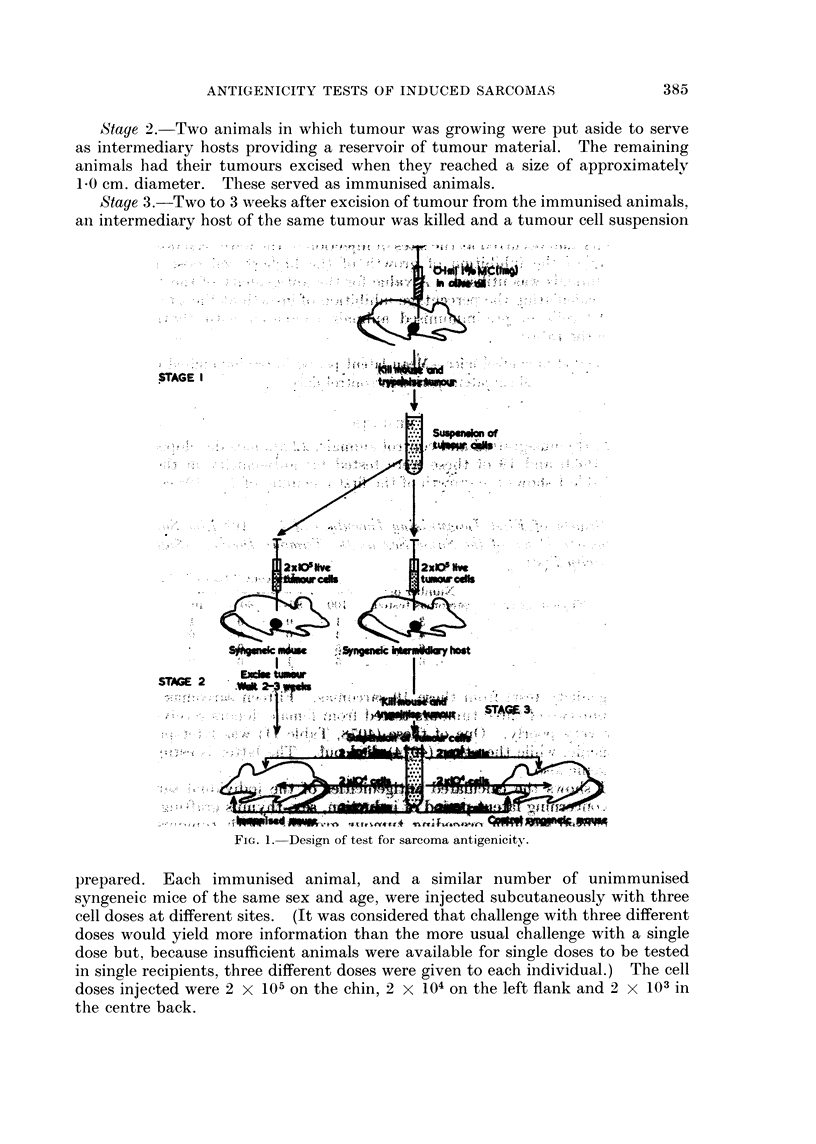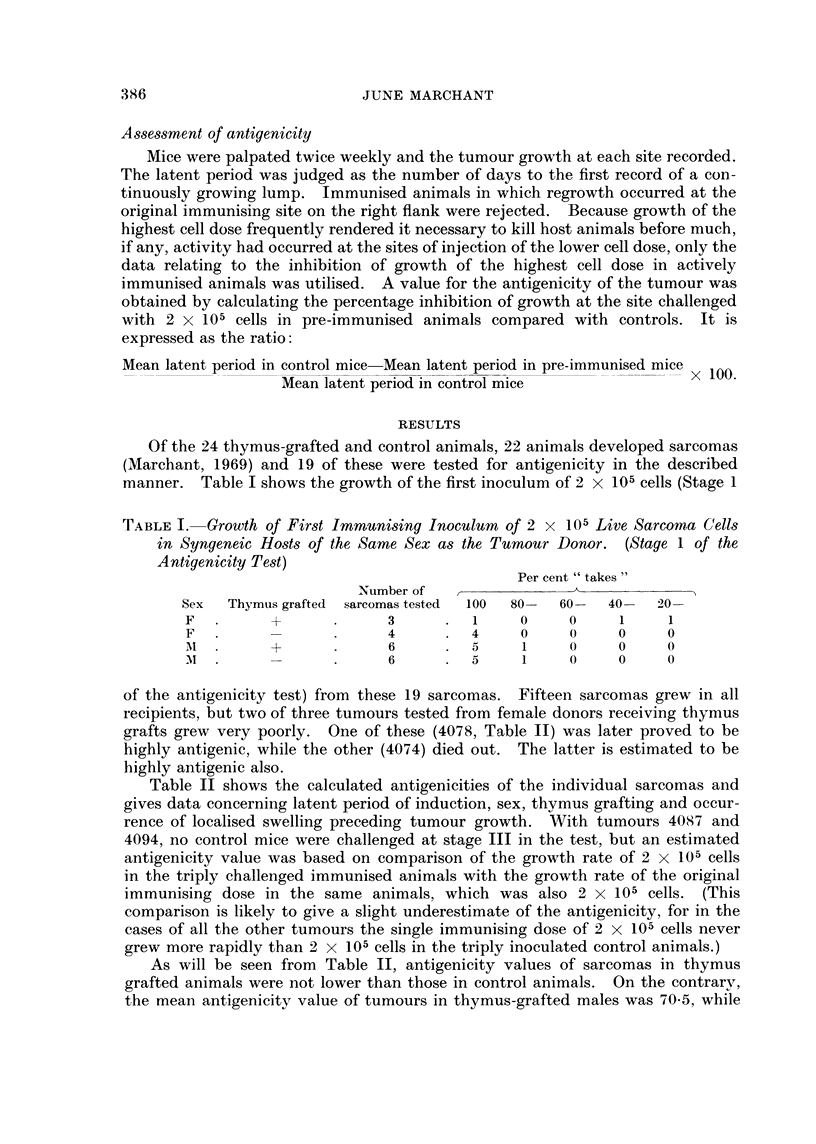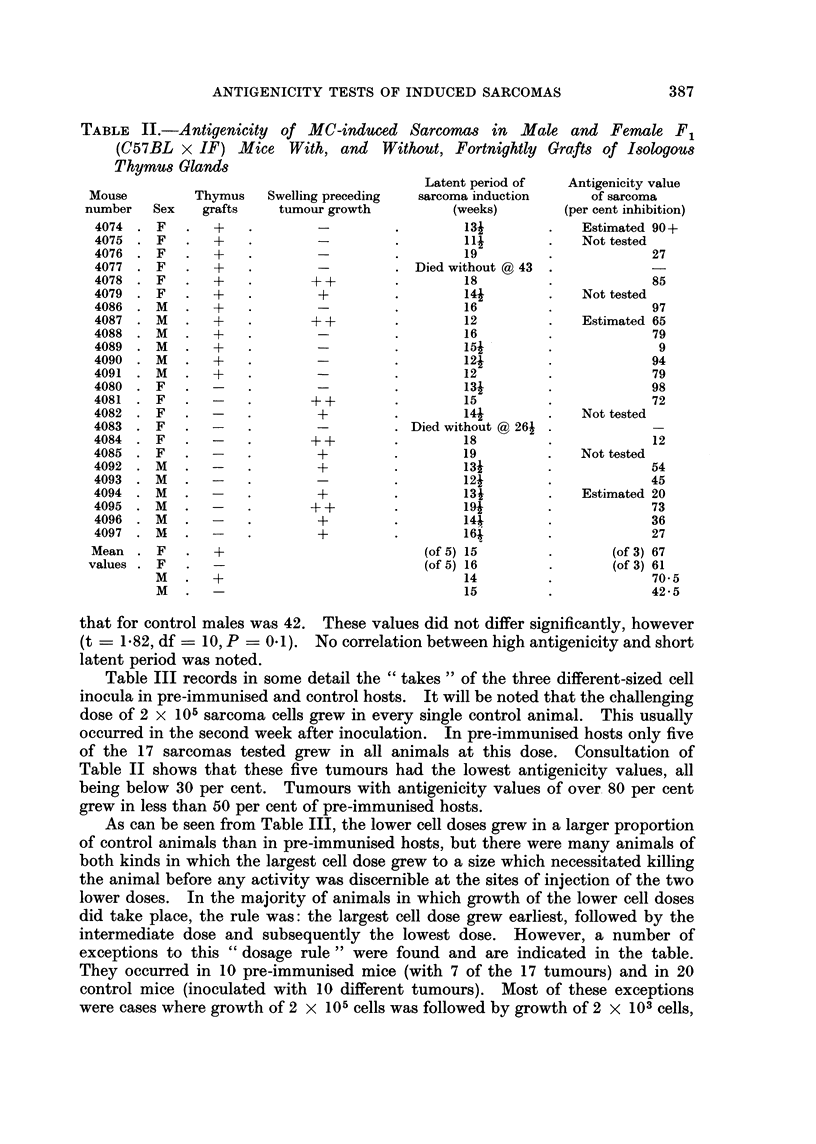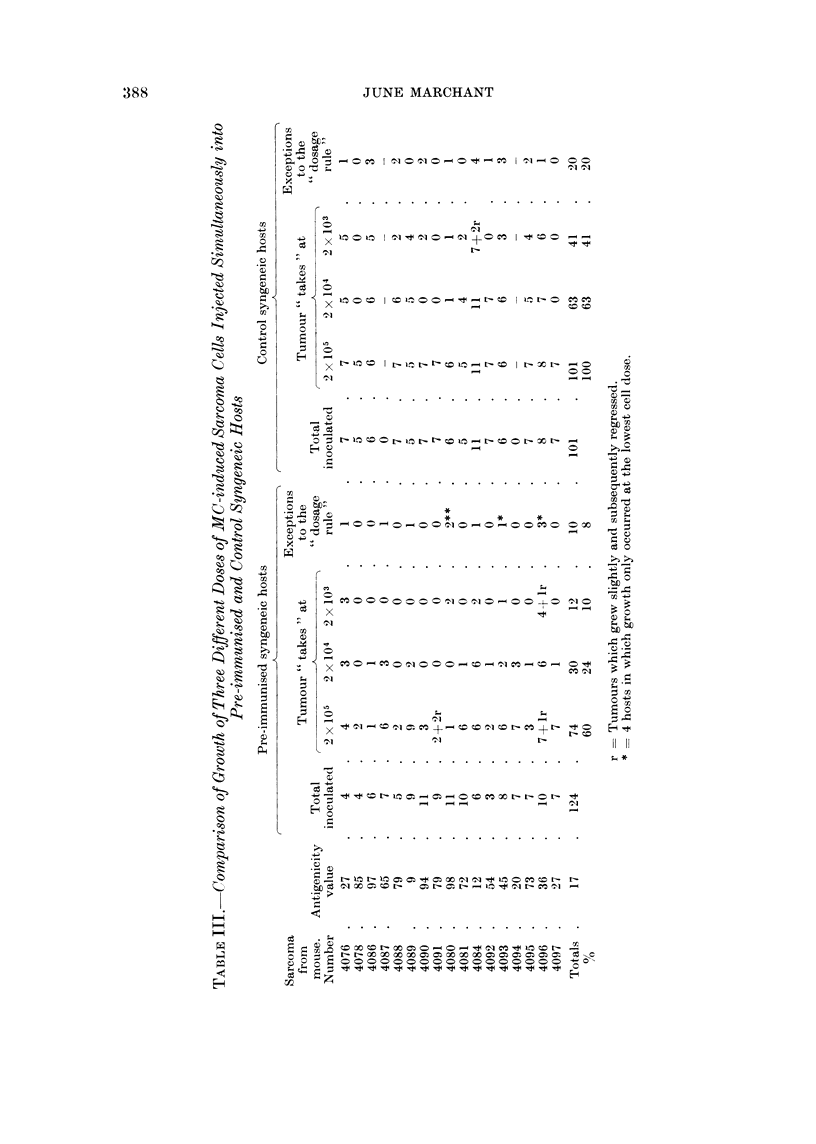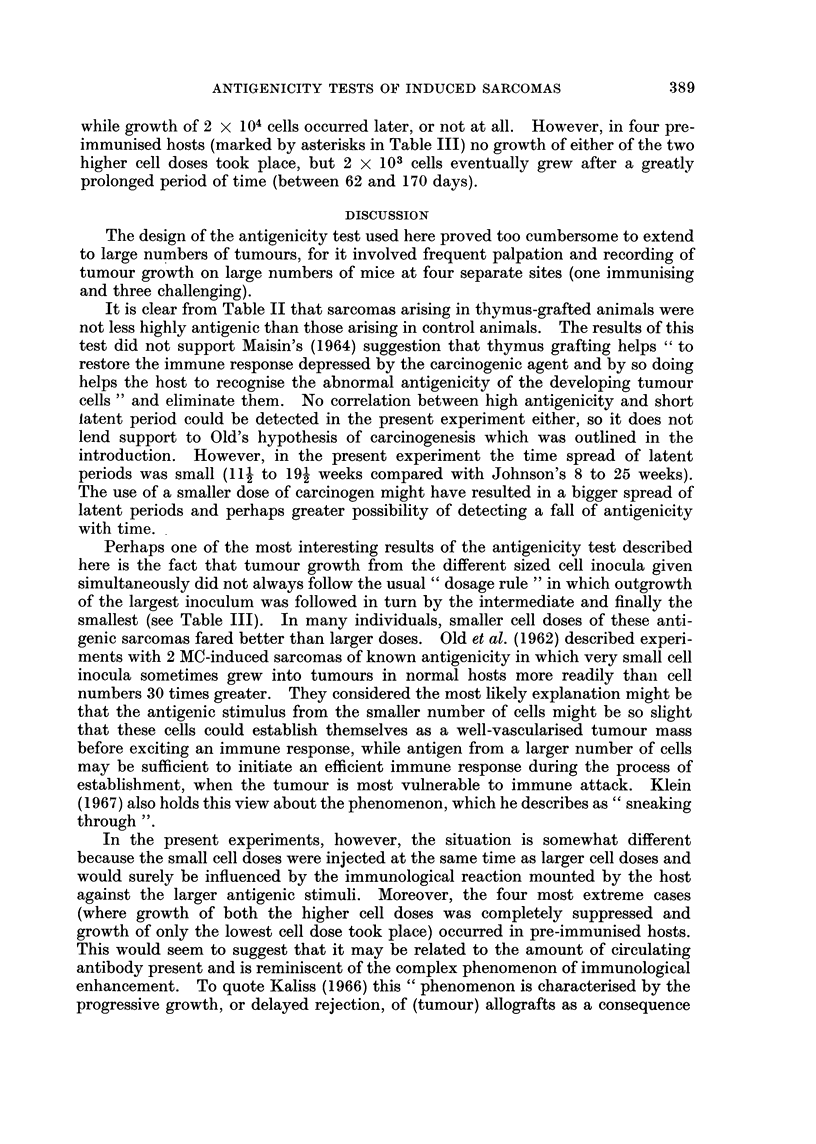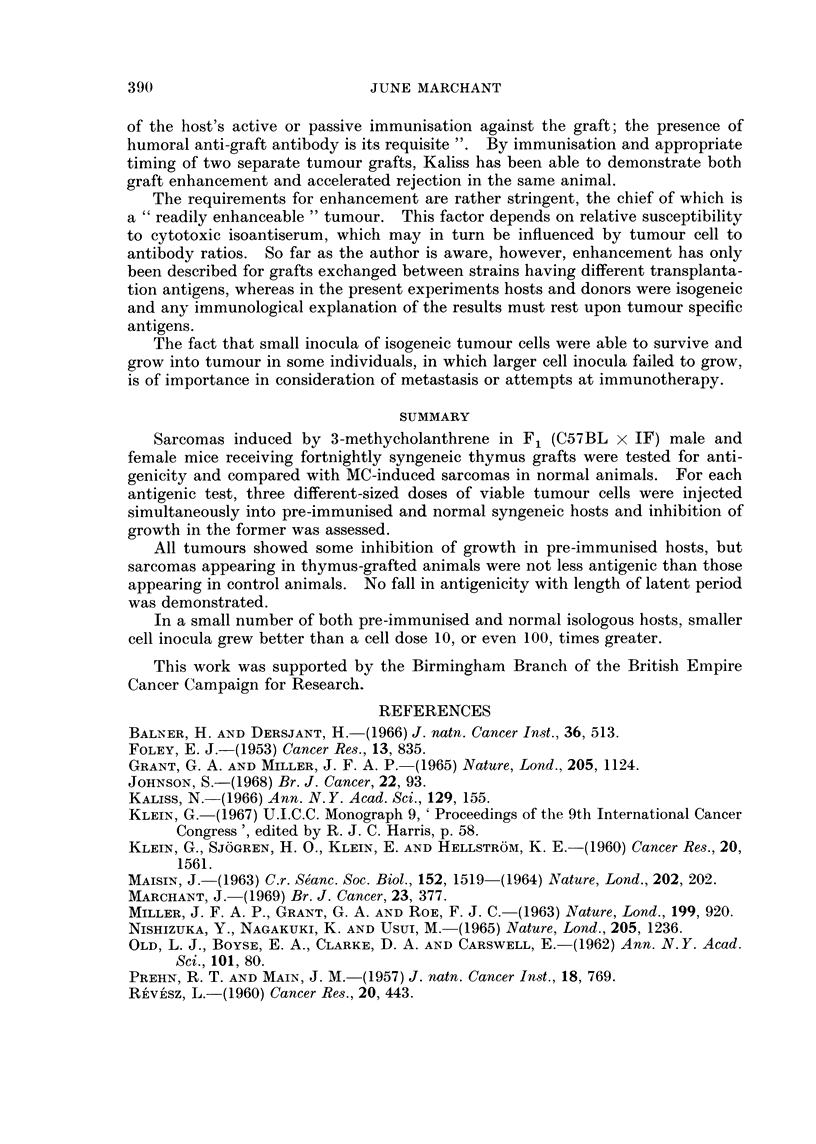# Sarcoma induction in mice by methylcholanthrene. Antigenicity tests of sarcomas induced in thymus grafted and control animals.

**DOI:** 10.1038/bjc.1969.50

**Published:** 1969-06

**Authors:** J. Marchant


### SARCOMA INDUCTION IN MICE BY METHYLCHOLANTHRENE

### ANTIGENICITY TESTS OF SARCOMAS INDUCED IN THYMUS GRAFTED AND CONTROL ANIMALS

JUNE MARCHANT

*From the Cancer Research Laboratories, Medical School, Birmingham* 15

Received for publication January 1, 1969

USING transplantation methods, several authors have shown that sarcomas induced by methylcholanthrene (MC) can elicit an immune response in syngeneic hosts (Foley, 1953; Prehn and Main, 1957; Révész, 1960; Old *et al.*, 1962), or even in the autochthonous host (Klein *et al.*, 1960). Immunity to the tumour is usually induced by transplanting the tumour to syngeneic hosts and allowing the animals to grow tumours which are then excised, or have their blood supply strangulated. Subsequent challenge of these pre-immunised hosts is made with viable cells from the same original tumour which has been maintained by passage in other syngeneic animals. The degree of antigenicity of a tumour can be measured by the difficulty with which immunity to it can be broken down.

Old *et al.* (1962), in a study of 11 MC-induced sarcomas found that the first four tumours to appear were highly antigenic, while some of those with longer latent periods had little or no demonstrable antigenicity. As a result of these findings, they proposed that the latent period of carcinogenesis is a selection period in which cells with neoplastic potential appear very early but, being so highly antigenic, they are promptly destroyed by the host. Tumours appear when the growth potential of the altered cells succeeds in over-riding any immunological response of the host. Later tumours, arising from cells which had been subjected to a longer period of immunological selection, would tend to be less antigenic.

If this immunological selection theory of MC carcinogenesis holds good, it would be expected that tumours arising in animals which had been subjected to long-term immune impairment might be more antigenic than those arising in normal animals. Two recent investigations have shown that early thymectomy of mice, which leads to permanent immunological impairment, does indeed cause the appearance of more highly antigenic sarcomas than in normal mice following MC injection (Balner and Dersjant, 1966; Johnson, 1968). Moreover, the latter author was able to show a decline in antigenicity of tumours as the latent period increased, in both intact and thymectomised animals.

The reports of Maisin (1963, 1964) that regular thymus grafting during the period of carcinogenesis appeared to increase the resistance of mice to skin tumour induction by MC, as opposed to the generally reported decrease of resistance to chemical induction of tumours in thymectomised animals (Miller *et al.*, 1963; Grant and Miller, 1965; Nishizuka *et al.*, 1965; Johnson, 1968), appeared to indicate that regular thymus grafting had the reverse effect of thymectomy on chemical carcinogenesis. Maisin considered that some kind of hormonal influence of the grafted thymus tissue helped to restore the immune response which had been depressed by the carcinogen and the host was thereby better able to recognise the abnormal antigenicity of developing tumour cells and to promote their immunological destruction. It follows from this line of reasoning that tumours arising in thymus-grafted (immune repaired) animals should be less antigenic than those arising in normal animals. Antigenic tests were therefore carried out on the MC-induced sarcomas in thymus-grafted and control animals which were the subject of the preceding communication (Marchant, 1969).

### MATERIALS AND METHODS

### *Tumours*

Male and female $F_1$ (C57BL $\times$ IF) mice were given a subcutaneous injection of 1 mg. of 3-methylcholanthrene (MC) in olive oil on the right flank when 3 months old. Half of the animals also received a subcutaneous graft of a whole thymus gland once a fortnight, beginning 5 weeks before the MC injection. The grafts came from 6- to 10-day-old syngeneic donors of the same sex. Sarcomas arose in 22 of 24 animals.

### *The antigenicity test*

For each tumour the test depended on a comparison of the growth of known numbers of viable tumour cells in actively immunised syngeneic hosts with the growth of similar doses in normal control animals.

### *Preparation of tumour cell suspensions*

All operations were carried out under sterile conditions. When the sarcomas were 1·0 to 2·0 cm. in diameter, the mice were killed and the tumours removed aseptically. They were minced with scissors and washed in Dulbecco " A " phosphate-buffered saline (Oxoid) with 100 $\mu$g. Streptomycin Sulphate B.P. (Glaxo) and 100 i.u. Sodium Benzyl Penicillin (Glaxo) per ml. The minced tumour was then transferred to a bottle containing a magnet and about 15 ml. 0·25 per cent trypsin (Difco) containing 0·2 mg. Deoxyribonuclease (BDH) per ml. It was then placed on a magnetic stirrer and incubated at 37° C. for half-an-hour, following which the suspended cells were removed and washed twice by gentle centrifugation. Cell clumps were sometimes removed by filtration through a fine web of glass wool. The number of viable cells in the final suspension was estimated in a haemocytometer by their ability to exclude eosin. The cell concentration was then adjusted to give the appropriate concentration of live tumour cells for subcutaneous injection, the volume of fluid injected being kept constant at 0·1 ml.

### *Design of the test*

The design of the test for antigenicity is represented in Fig. 1. (Three tumours which were growing very poorly in their original hosts were passaged once before the test was commenced, in order to obtain sufficient material.)

*Stage* 1.—About 6 to 12 syngeneic mice of the same sex as the tumour donor were injected subcutaneously on the right flank with $2 \times 10^5$ live tumour cells in 0·1 ml. saline.

*Stage* 2.—Two animals in which tumour was growing were put aside to serve as intermediary hosts providing a reservoir of tumour material. The remaining animals had their tumours excised when they reached a size of approximately 1·0 cm. diameter. These served as immunised animals.

*Stage* 3.—Two to 3 weeks after excision of tumour from the immunised animals, an intermediary host of the same tumour was killed and a tumour cell suspension

FIG. 1.—Design of test for sarcoma antigenicity.

prepared. Each immunised animal, and a similar number of unimmunised syngeneic mice of the same sex and age, were injected subcutaneously with three cell doses at different sites. (It was considered that challenge with three different doses would yield more information than the more usual challenge with a single dose but, because insufficient animals were available for single doses to be tested in single recipients, three different doses were given to each individual.) The cell doses injected were $2 \times 10^5$ on the chin, $2 \times 10^4$ on the left flank and $2 \times 10^3$ in the centre back.

*Assessment of antigenicity*

Mice were palpated twice weekly and the tumour growth at each site recorded. The latent period was judged as the number of days to the first record of a continuously growing lump. Immunised animals in which regrowth occurred at the original immunising site on the right flank were rejected. Because growth of the highest cell dose frequently rendered it necessary to kill host animals before much, if any, activity had occurred at the sites of injection of the lower cell dose, only the data relating to the inhibition of growth of the highest cell dose in actively immunised animals was utilised. A value for the antigenicity of the tumour was obtained by calculating the percentage inhibition of growth at the site challenged with $2 \times 10^5$ cells in pre-immunised animals compared with controls. It is expressed as the ratio:

$$\frac{\text{Mean latent period in control mice—Mean latent period in pre-immunised mice}}{\text{Mean latent period in control mice}} \times 100.$$

RESULTS

Of the 24 thymus-grafted and control animals, 22 animals developed sarcomas (Marchant, 1969) and 19 of these were tested for antigenicity in the described manner. Table I shows the growth of the first inoculum of $2 \times 10^5$ cells (Stage 1 of the antigenicity test) from these 19 sarcomas. Fifteen sarcomas grew in all recipients, but two of three tumours tested from female donors receiving thymus grafts grew very poorly. One of these (4078, Table II) was later proved to be highly antigenic, while the other (4074) died out. The latter is estimated to be highly antigenic also.

TABLE I.—*Growth of First Immunising Inoculum of* $2 \times 10^5$ *Live Sarcoma Cells in Syngeneic Hosts of the Same Sex as the Tumour Donor. (Stage* 1 *of the Antigenicity Test)*

| Sex | Thymus grafted | Number of sarcomas tested | Per cent " takes " | | | | |
|---|---|---|---|---|---|---|---|
| | | | 100 | 80— | 60— | 40— | 20— |
| F | + | 3 | 1 | 0 | 0 | 1 | 1 |
| F | — | 4 | 4 | 0 | 0 | 0 | 0 |
| M | + | 6 | 5 | 1 | 0 | 0 | 0 |
| M | — | 6 | 5 | 1 | 0 | 0 | 0 |

Table II shows the calculated antigenicities of the individual sarcomas and gives data concerning latent period of induction, sex, thymus grafting and occurrence of localised swelling preceding tumour growth. With tumours 4087 and 4094, no control mice were challenged at stage III in the test, but an estimated antigenicity value was based on comparison of the growth rate of $2 \times 10^5$ cells in the triply challenged immunised animals with the growth rate of the original immunising dose in the same animals, which was also $2 \times 10^5$ cells. (This comparison is likely to give a slight underestimate of the antigenicity, for in the cases of all the other tumours the single immunising dose of $2 \times 10^5$ cells never grew more rapidly than $2 \times 10^5$ cells in the triply inoculated control animals.)

As will be seen from Table II, antigenicity values of sarcomas in thymus grafted animals were not lower than those in control animals. On the contrary, the mean antigenicity value of tumours in thymus-grafted males was 70·5, while

TABLE II.—*Antigenicity of MC-induced Sarcomas in Male and Female $F_1$ (C57BL × IF) Mice With, and Without, Fortnightly Grafts of Isologous Thymus Glands*

| Mouse number | Sex | Thymus grafts | Swelling preceding tumour growth | Latent period of sarcoma induction (weeks) | Antigenicity value of sarcoma (per cent inhibition) |
|---|---|---|---|---|---|
| 4074 | F | + | — | 13½ | Estimated 90+ |
| 4075 | F | + | — | 11½ | Not tested |
| 4076 | F | + | — | 19 | 27 |
| 4077 | F | + | — | Died without @ 43 | — |
| 4078 | F | + | ++ | 18 | 85 |
| 4079 | F | + | + | 14½ | Not tested |
| 4086 | M | + | — | 16 | 97 |
| 4087 | M | + | ++ | 12 | Estimated 65 |
| 4088 | M | + | — | 16 | 79 |
| 4089 | M | + | — | 15½ | 9 |
| 4090 | M | + | — | 12½ | 94 |
| 4091 | M | + | — | 12 | 79 |
| 4080 | F | — | — | 13½ | 98 |
| 4081 | F | — | ++ | 15 | 72 |
| 4082 | F | — | + | 14½ | Not tested |
| 4083 | F | — | — | Died without @ 26½ | — |
| 4084 | F | — | ++ | 18 | 12 |
| 4085 | F | — | + | 19 | Not tested |
| 4092 | M | — | + | 13½ | 54 |
| 4093 | M | — | — | 12½ | 45 |
| 4094 | M | — | + | 13½ | Estimated 20 |
| 4095 | M | — | ++ | 19½ | 73 |
| 4096 | M | — | + | 14½ | 36 |
| 4097 | M | — | + | 16½ | 27 |
| Mean values | F | + | | (of 5) 15 | (of 3) 67 |
| | F | — | | (of 5) 16 | (of 3) 61 |
| | M | + | | 14 | 70·5 |
| | M | — | | 15 | 42·5 |

that for control males was 42. These values did not differ significantly, however ($t = 1{\cdot}82$, $df = 10$, $P = 0{\cdot}1$). No correlation between high antigenicity and short latent period was noted.

Table III records in some detail the " takes " of the three different-sized cell inocula in pre-immunised and control hosts. It will be noted that the challenging dose of $2 \times 10^5$ sarcoma cells grew in every single control animal. This usually occurred in the second week after inoculation. In pre-immunised hosts only five of the 17 sarcomas tested grew in all animals at this dose. Consultation of Table II shows that these five tumours had the lowest antigenicity values, all being below 30 per cent. Tumours with antigenicity values of over 80 per cent grew in less than 50 per cent of pre-immunised hosts.

As can be seen from Table III, the lower cell doses grew in a larger proportion of control animals than in pre-immunised hosts, but there were many animals of both kinds in which the largest cell dose grew to a size which necessitated killing the animal before any activity was discernible at the sites of injection of the two lower doses. In the majority of animals in which growth of the lower cell doses did take place, the rule was: the largest cell dose grew earliest, followed by the intermediate dose and subsequently the lowest dose. However, a number of exceptions to this " dosage rule " were found and are indicated in the table. They occurred in 10 pre-immunised mice (with 7 of the 17 tumours) and in 20 control mice (inoculated with 10 different tumours). Most of these exceptions were cases where growth of $2 \times 10^5$ cells was followed by growth of $2 \times 10^3$ cells,

TABLE III.—*Comparison of Growth of Three Different Doses of MC-induced Sarcoma Cells Injected Simultaneously into Pre-immunised and Control Syngeneic Hosts*

| | | Pre-immunised syngeneic hosts | | | | | Control syngeneic hosts | | | | |
|---|---|---|---|---|---|---|---|---|---|---|---|
| | | | Tumour " takes " at | | | | | Tumour " takes " at | | | |
| Sarcoma from mouse. Number | Antigenicity value | Total inoculated | $2 \times 10^5$ | $2 \times 10^4$ | $2 \times 10^3$ | Exceptions to the " dosage rule " | Total inoculated | $2 \times 10^5$ | $2 \times 10^4$ | $2 \times 10^3$ | Exceptions to the " dosage rule " |
| 4076 | 27 | 4 | 4 | 3 | 3 | 1 | 7 | 7 | 5 | 5 | 1 |
| 4078 | 85 | 4 | 2 | 0 | 0 | 0 | 5 | 5 | 0 | 0 | 0 |
| 4086 | 97 | 6 | 1 | 1 | 0 | 0 | 6 | 6 | 6 | 5 | 3 |
| 4087 | 65 | 7 | 6 | 3 | 0 | 1 | 0 | – | – | – | – |
| 4088 | 79 | 5 | 2 | 0 | 0 | 0 | 7 | 7 | 6 | 2 | 2 |
| 4089 | 9 | 9 | 9 | 2 | 0 | 1 | 5 | 5 | 5 | 4 | 0 |
| 4090 | 94 | 11 | 3 | 0 | 0 | 0 | 7 | 7 | 0 | 2 | 2 |
| 4091 | 79 | 9 | 2+2r | 0 | 0 | 0 | 7 | 7 | 0 | 0 | 0 |
| 4080 | 98 | 11 | 1 | 0 | 2 | 2** | 6 | 6 | 1 | 1 | 1 |
| 4081 | 72 | 10 | 6 | 1 | 0 | 0 | 5 | 5 | 4 | 2 | 0 |
| 4084 | 12 | 6 | 6 | 6 | 2 | 1 | 11 | 11 | 11 | 7+2r | 4 |
| 4092 | 54 | 3 | 2 | 1 | 0 | 0 | 7 | 7 | 7 | 0 | 1 |
| 4093 | 45 | 8 | 6 | 2 | 1 | 1* | 6 | 6 | 6 | 3 | 3 |
| 4094 | 20 | 7 | 7 | 3 | 0 | 0 | 0 | – | – | – | – |
| 4095 | 73 | 7 | 3 | 1 | 0 | 0 | 7 | 7 | 5 | 4 | 2 |
| 4096 | 36 | 10 | 7+1r | 6 | 4+1r | 3* | 8 | 8 | 7 | 6 | 1 |
| 4097 | 27 | 7 | 7 | 1 | 0 | 0 | 7 | 7 | 0 | 0 | 0 |
| Totals | 17 | 124 | 74 | 30 | 12 | 10 | 101 | 101 | 63 | 41 | 20 |
| % | | | 60 | 24 | 10 | 8 | | 100 | 63 | 41 | 20 |

r = Tumours which grew slightly and subsequently regressed.

* = 4 hosts in which growth only occurred at the lowest cell dose.

while growth of $2 \times 10^4$ cells occurred later, or not at all. However, in four pre-immunised hosts (marked by asterisks in Table III) no growth of either of the two higher cell doses took place, but $2 \times 10^3$ cells eventually grew after a greatly prolonged period of time (between 62 and 170 days).

### DISCUSSION

The design of the antigenicity test used here proved too cumbersome to extend to large numbers of tumours, for it involved frequent palpation and recording of tumour growth on large numbers of mice at four separate sites (one immunising and three challenging).

It is clear from Table II that sarcomas arising in thymus-grafted animals were not less highly antigenic than those arising in control animals. The results of this test did not support Maisin's (1964) suggestion that thymus grafting helps " to restore the immune response depressed by the carcinogenic agent and by so doing helps the host to recognise the abnormal antigenicity of the developing tumour cells " and eliminate them. No correlation between high antigenicity and short latent period could be detected in the present experiment either, so it does not lend support to Old's hypothesis of carcinogenesis which was outlined in the introduction. However, in the present experiment the time spread of latent periods was small ($11\frac{1}{2}$ to $19\frac{1}{2}$ weeks compared with Johnson's 8 to 25 weeks). The use of a smaller dose of carcinogen might have resulted in a bigger spread of latent periods and perhaps greater possibility of detecting a fall of antigenicity with time.

Perhaps one of the most interesting results of the antigenicity test described here is the fact that tumour growth from the different sized cell inocula given simultaneously did not always follow the usual " dosage rule " in which outgrowth of the largest inoculum was followed in turn by the intermediate and finally the smallest (see Table III). In many individuals, smaller cell doses of these antigenic sarcomas fared better than larger doses. Old *et al.* (1962) described experiments with 2 MC-induced sarcomas of known antigenicity in which very small cell inocula sometimes grew into tumours in normal hosts more readily than cell numbers 30 times greater. They considered the most likely explanation might be that the antigenic stimulus from the smaller number of cells might be so slight that these cells could establish themselves as a well-vascularised tumour mass before exciting an immune response, while antigen from a larger number of cells may be sufficient to initiate an efficient immune response during the process of establishment, when the tumour is most vulnerable to immune attack. Klein (1967) also holds this view about the phenomenon, which he describes as " sneaking through ".

In the present experiments, however, the situation is somewhat different because the small cell doses were injected at the same time as larger cell doses and would surely be influenced by the immunological reaction mounted by the host against the larger antigenic stimuli. Moreover, the four most extreme cases (where growth of both the higher cell doses was completely suppressed and growth of only the lowest cell dose took place) occurred in pre-immunised hosts. This would seem to suggest that it may be related to the amount of circulating antibody present and is reminiscent of the complex phenomenon of immunological enhancement. To quote Kaliss (1966) this " phenomenon is characterised by the progressive growth, or delayed rejection, of (tumour) allografts as a consequence of the host's active or passive immunisation against the graft; the presence of humoral anti-graft antibody is its requisite ". By immunisation and appropriate timing of two separate tumour grafts, Kaliss has been able to demonstrate both graft enhancement and accelerated rejection in the same animal.

The requirements for enhancement are rather stringent, the chief of which is a " readily enhanceable " tumour. This factor depends on relative susceptibility to cytotoxic isoantiserum, which may in turn be influenced by tumour cell to antibody ratios. So far as the author is aware, however, enhancement has only been described for grafts exchanged between strains having different transplantation antigens, whereas in the present experiments hosts and donors were isogeneic and any immunological explanation of the results must rest upon tumour specific antigens.

The fact that small inocula of isogeneic tumour cells were able to survive and grow into tumour in some individuals, in which larger cell inocula failed to grow, is of importance in consideration of metastasis or attempts at immunotherapy.

### SUMMARY

Sarcomas induced by 3-methycholanthrene in $F_1$ (C57BL $\times$ IF) male and female mice receiving fortnightly syngeneic thymus grafts were tested for antigenicity and compared with MC-induced sarcomas in normal animals. For each antigenic test, three different-sized doses of viable tumour cells were injected simultaneously into pre-immunised and normal syngeneic hosts and inhibition of growth in the former was assessed.

All tumours showed some inhibition of growth in pre-immunised hosts, but sarcomas appearing in thymus-grafted animals were not less antigenic than those appearing in control animals. No fall in antigenicity with length of latent period was demonstrated.

In a small number of both pre-immunised and normal isologous hosts, smaller cell inocula grew better than a cell dose 10, or even 100, times greater.

This work was supported by the Birmingham Branch of the British Empire Cancer Campaign for Research.

### REFERENCES

Balner, H. and Dersjant, H.—(1966) *J. natn. Cancer Inst.*, **36**, 513.
Foley, E. J.—(1953) *Cancer Res.*, **13**, 835.
Grant, G. A. and Miller, J. F. A. P.—(1965) *Nature, Lond.*, **205**, 1124.
Johnson, S.—(1968) *Br. J. Cancer*, **22**, 93.
Kaliss, N.—(1966) *Ann. N.Y. Acad. Sci.*, **129**, 155.
Klein, G.—(1967) U.I.C.C. Monograph 9, ' Proceedings of the 9th International Cancer Congress ', edited by R. J. C. Harris, p. 58.
Klein, G., Sjögren, H. O., Klein, E. and Hellström, K. E.—(1960) *Cancer Res.*, **20**, 1561.
Maisin, J.—(1963) *C.r. Séanc. Soc. Biol.*, **152**, 1519—(1964) *Nature, Lond.*, **202**, 202.
Marchant, J.—(1969) *Br. J. Cancer*, **23**, 377.
Miller, J. F. A. P., Grant, G. A. and Roe, F. J. C.—(1963) *Nature, Lond.*, **199**, 920.
Nishizuka, Y., Nagakuki, K. and Usui, M.—(1965) *Nature, Lond.*, **205**, 1236.
Old, L. J., Boyse, E. A., Clarke, D. A. and Carswell, E.—(1962) *Ann. N.Y. Acad. Sci.*, **101**, 80.
Prehn, R. T. and Main, J. M.—(1957) *J. natn. Cancer Inst.*, **18**, 769.
Révész, L.—(1960) *Cancer Res.*, **20**, 443.